# The Role of Resilience in Coping With Future Uncertainty Among People With Brain Tumors: Cross-Sectional Study

**DOI:** 10.2196/71674

**Published:** 2026-01-06

**Authors:** Li-Ting Huang Longcoy, Shu-Yuan Liang, Ardith Z Doorenbos

**Affiliations:** 1 College of Nursing University of Illinois Chicago Chicago, IL United States; 2 National Taipei University of Nursing and Health Sciences Taipei City Taiwan

**Keywords:** resilience, problem-focused coping, emotion-focused coping, adaptation, uncertainty in illness theory

## Abstract

**Background:**

Adults with brain tumors learn to navigate unpredictable physical and psychological symptoms along with the possibilities of tumor recurrence. As a result, they tend to become resilient to confronting profound uncertainty and actively employ coping strategies. Yet, the impact of resilience on coping strategies among people with brain tumors has not been fully explored.

**Objective:**

This study aimed to examine the effects of resilience on the association between future uncertainty and two distinct types of coping strategies (problem-focused coping and emotion-focused coping) among people with brain tumors in Taiwan.

**Methods:**

A parent study recruited 95 adults with brain tumors undergoing at least 1 month of chemotherapy or radiotherapy at a veterans general hospital in northern Taiwan. We assessed resilience, future uncertainty, and coping strategies via a secondary analysis of data from the parent study collected using the Chinese versions of the Resilience Scale, the European Organization for Research and Treatment of Cancer’s Quality of Life Questionnaire for brain cancer, and the revised Ways of Coping Checklist. Simple mediation models were conducted to examine the role of resilience between future uncertainty and the two types of coping strategies.

**Results:**

Most participants demonstrated low resilience and responded to stress with both problem- and emotion-focused coping strategies. Simple mediation analyses showed a statistically significant association between an increase in resilience and adoption of each type of coping strategy. In addition, resilience was a statistically significant mediator in the association between future uncertainty and both problem- and emotion-focused coping strategies.

**Conclusions:**

Brain tumor disease trajectories require people to effectively adopt both problem- and emotion-focused coping strategies to confront uncertainty. Health care providers play a crucial role in evaluating and fostering their patients’ resilience to promote adaptability through effective coping strategies.

## Introduction

Given the nature of brain tumors, uncertainty may persist throughout the cancer trajectory. In Taiwan, brain tumors account for 1.2% of cancer cases; however, the mortality rate has been rising for 2 decades [1]. The lack of clarity about prognoses leads people with brain tumors to experience ongoing uncertainty along with unpredictable symptom patterns and increases in functional dependency [2]. As a result, these sources of uncertainty may negatively affect their ability to cope with cancer [3].

According to Mishel’s uncertainty in illness theory [4], an individual responds to uncertainty by assessing whether it presents a danger or an opportunity. When there is a potential positive outcome from the uncertainty, the individual may use strategies such as selective ignoring to maintain uncertainty as a source of hope. A danger appraisal may prompt the individual to take action to mitigate the uncertainty [4,5]. For people with brain tumors, the ongoing perception of uncertainty highlights the need for effective coping strategies to support successful adaptation; this is a process that may be strengthened through resilience. The known positive association between resilience and cancer coping [6,7] suggests that cultivating resilience may enhance cognitive appraisal processes and thus increase the adoption of effective coping strategies.

Resilience is defined as the process by which an individual can, in the face of stress, flexibly utilize available external resources (eg, social support from others), internal resources (eg, individual strengths and skills), and existential resources (eg, practices of meaning-making and expressing gratitude) [8]. A qualitative study of people with brain tumors identified the need for resilience, including living in the moment and finding joy, as coping mechanisms for navigating uncertainty [9]. Other studies have shown that resilience mediates the association between symptom distress and quality of life and that resilience has effects on reducing psychological distress [10]. These findings highlight that resilience can serve as a protective factor to empower people to demonstrate flexibility in overcoming difficulties through various resources and coping strategies.

There has been little research on the impact of resilience on how individuals with brain tumors cope with uncertainty. Thus, this study, guided by the uncertainty in illness theory, investigates the effects of resilience on the association between uncertainty and individual coping strategies, including problem-focused coping and emotion-focused coping, among people with brain tumors.

## Methods

### Sample and Setting

This secondary analysis used data from 95 people with primary brain tumors, which was sufficient to reach the desired power of .80 to examine the effect of future uncertainty on coping strategies through resilience. Participants were recruited during an outpatient visit to a veterans general hospital in Taiwan. Eligibility criteria were (1) patients aged 20 years or older and (2) patients diagnosed with a benign or malignant primary brain tumor and receiving at least 1 month of treatment. People with a diagnosed mental illness or unable to communicate were not eligible to participate.

### Ethical Considerations

The parent study was approved by the Taipei Veterans General Hospital (2014-09-007AC) institutional review board (for more details about the parent study, please see [11,12]). Before consenting, participants were informed that they could withdraw from the study at any time without penalty, and that this would not affect their treatment. In addition, no identifiable health information was collected or included in the statistical or data management programs. No compensation was provided to participants for completing the survey.

### Instruments

#### Coping Strategies

We used the Chinese version of the Ways of Coping Checklist–Revised to assess participants’ coping strategies. The Ways of Coping Checklist–Revised uses 42 items to assess 5 factors: problem-focused coping, seeking social support, self-blame, distancing, and wishful thinking [13]. We categorized the first 2 factors as problem-focused coping and the other 3 factors as emotion-focused coping [13]. All items use a 5-point Likert scale (0 to 4); higher scores indicate more frequent adoption of a strategy. The McDonald ω for the internal consistency of the total scores of problem-focused and emotion-focused coping were 0.86 and 0.83, respectively.

#### Future Uncertainty

Participants’ future uncertainty was measured using the subscale of the European Organization for Research and Treatment of Cancer’s Quality of Life Questionnaire [14]. The 4 items that measure future uncertainty are as follows: (1) Did you feel uncertain about the future? (2) Did you feel you had setbacks in your condition? (3) Were you concerned about disruption of family life? and (4) Did your outlook on the future worsen? All items were measured on a scale of 1 to 4; higher scores represent worse symptoms. The McDonald ω for the total score was 0.80.

#### Resilience

Participants’ resilience was assessed using the Chinese version of the Resilience Scale, with 25 items in 5 domains of resilience, including meaningful life, perseverance, self-reliance, equanimity, and existential aloneness. All items use a 7-point Likert scale ranging from 1 to 7, for a total score ranging from 25 to 175 (≥146=moderately high resilience; 131-145=moderate resilience; ≤130=low resilience) [15]. The McDonald ω for the total score was 0.97.

### Data Analysis

To examine how resilience affects the effect of future uncertainty on coping, we conducted 2 simple mediation models, with resilience as the mediator and future uncertainty as the predictor of the two different outcome variables: problem-focused coping and emotion-focused coping. Each model consisted of one direct effect of future uncertainty (X) as a predictor of coping strategies (Y) and one indirect effect of future uncertainty (X) on coping strategies (Y) through resilience (M). Inferences about direct and indirect effects were estimated by bootstrapping, which generated 95% percentile bootstrap CIs [16].

## Results

Participants adopted both problem-focused coping strategies (mean 58.06, SD 11.10) and emotion-focused coping strategies (mean 43.96, SD 12.06). Half (48/95, 51%) of the participants reported low resilience, and a quarter each reported moderate (24/95, 25%) and moderately high (23/95, 24%) resilience.

**Figure 1:**
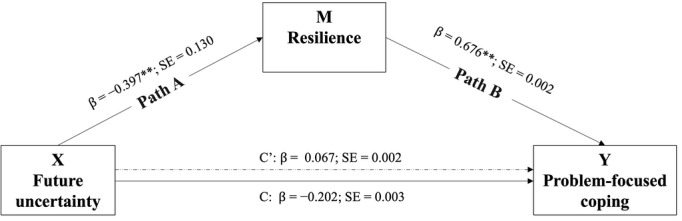
Simple mediation model estimation results. C' is the direct effect of future uncertainty on problem-focused coping; C is the total effect of future uncertainty on problem-focused coping. *P<.05; **P<.01.

Two simple mediation models examined the role of resilience in the different types of coping strategies. The model for resilience as a mediator between future uncertainty and problem-focused coping resulted in a standardized coefficient of total effect of −0.202 (Figure 1). When the total effect of future uncertainty was partitioned into direct and indirect effects, the indirect effect of future uncertainty on problem-focused coping through resilience (Figure 1, path A × path B) was statistically different from zero (β=−0.268, SE 0.068, 95% CI −0.406 to −0.142).

**Figure 2:**
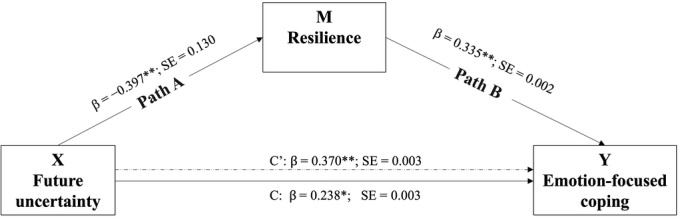
Simple mediation model estimation results. C' is the direct effect of future uncertainty on emotion-focused coping; C is the total effect of future uncertainty on emotion-focused coping. *P<.05; **P<.01.

The model with emotion-focused coping as the outcome resulted in a total effect of future uncertainty that was statistically significant (Figure 2). The direct effect of future uncertainty on emotion-focused coping was 0.370. The indirect effect of uncertainty through resilience was significantly different from zero (β=−0.133, SE 0.053, 95% CI −0.249 to −0.041).

## Discussion

### Principal Findings

This study investigates the influence of resilience on adopting 2 distinct coping strategies amidst the uncertainty associated with brain tumors. We found that resilience positively facilitated the adoption of both problem-focused and emotion-focused coping strategies and a mediating role for resilience between uncertainty and individual coping strategies. In particular, the mediating effects of resilience appeared to be more pronounced for problem-focused coping, as the direct effect of uncertainty on problem-focused coping became nonsignificant when resilience was included. Indeed, focus group discussions among people with brain tumors revealed that constant uncertainty led to fear, despair, and adjustment difficulties. Over time, they learned to adopt problem-focused coping strategies such as planning end-of-life care to reduce caregiver burden [9]. In other words, resilience is a process of realization through awareness of accurate appraisals, available resources, and the flexible use of coping strategies [8]. When the cause of uncertainty can be managed, awareness of available informational and instrumental social support through resilience may increase the likelihood of adopting problem-focused coping strategies with greater confidence [7,9,17].

Unclear symptom patterns and prognoses for brain tumors may prompt people to adopt emotion-focused coping strategies. People with brain tumors expressed the need to maintain hope as part of their coping strategies and to live in the present moment to mitigate the impact of psychological distress [9]. In fact, cultivating hope is integral to developing resilience [18], and it has been identified as a predictor of resilience among individuals with cancer [6]. Interestingly, our findings showed that the reduction in resilience resulting from increased uncertainty weakened the overall effect of uncertainty on emotion-focused coping. One possible explanation is that in coming to terms with their condition, people with brain tumors may ultimately cultivate hope, which in turn strengthens resilience and enables them to flexibly adopt either problem-focused or emotion-focused coping strategies in response to uncertainty.

### Limitations

This study captured participants’ resilience levels at only one point in time. Our goal was to examine the impact of resilience while recruiting a sufficient sample for analysis within a feasible recruitment period. The study’s sample size may limit further investigation. Although our sample shows slightly lower resilience, it remains generally comparable to other samples with brain tumors [19,20].

### Clinical Implications

Health care providers can help patients with brain tumors develop resilience by recognizing and applying their external, internal, and existential resources to address uncertainty. With such assistance, the process of building resilience can be accelerated within 12 weeks [21]. Additionally, health care providers can provide meaning-making or gratitude exercises to increase resilience and facilitate successful adaptation [22].

### Conclusion

This study highlights the importance of developing resilience to help people with brain tumors accurately appraise and flexibly apply effective coping strategies. The findings provide potential targets for a resilience-building intervention to reduce the impact of uncertainty.

## Data Availability

The data collected during this study are available from the corresponding author upon reasonable request.
